# Pyruvate kinase M2 is a poor prognostic marker of and a therapeutic target in ovarian cancer

**DOI:** 10.1371/journal.pone.0182166

**Published:** 2017-07-28

**Authors:** Tai-Kuang Chao, Tien-Shuo Huang, Yu-Ping Liao, Rui-Lan Huang, Po-Hsuan Su, Hueng-Yuan Shen, Hung-Cheng Lai, Yu-Chi Wang

**Affiliations:** 1 Graduate Institute of Medical Sciences, National Defense Medical Center, Taipei, Taiwan; 2 Department of Pathology, Tri-Service General Hospital, National Defense Medical Centre, Taipei, Taiwan; 3 Laboratory of Epigenetics and Cancer Stem Cells, National Defense Medical Centre, Taipei, Taiwan; 4 Department of Obstetrics and Gynecology, Shuang-Ho Hospital, Taipei Medical University, New Taipei City, Taiwan; 5 Department of Obstetrics and Gynecology, School of medicine, College of medicine, Taipei Medical University, Taipei, Taiwan; 6 Department of Nuclear Medicine and PET center, Tri-Service General Hospital, National Defense Medical Centre, Taipei, Taiwan; 7 Department of Obstetrics and Gynecology, Tri-Service General Hospital, National Defense Medical Centre, Taipei, Taiwan; University of Nebraska Medical Center, UNITED STATES

## Abstract

Pyruvate kinase M2 (PKM2) regulates glycolysis and oxidative phosphorylation; however, the role of PKM2 in ovarian cancer remains largely unknown. We investigated whether ovarian cancer metabolism could provide insight into the development of therapeutic strategies. We performed immunohistochemical staining for PKM2 on a tissue microarray for multivariate analysis. It revealed that patients exhibiting higher PKM2 expression were significantly associated with malignancy groups (*p <* 0.001) and pathogenesis models (*p <* 0.001), had poor progression-free survival rates (*p* = 0.01) as compared with patients exhibiting lower PKM2 levels, and yielded a hazard ratio of death of 2.02 (95% confidence interval: 0.70–5.85). In cell lines, PKM2 inhibitor significantly inhibited the glycolytic rate according to cellular glucose consumption (*p* < 0.001). We also utilized Seahorse assays to assess metabolism-related cell-specific factors and the impact of PKM2 inhibitors. Energy shifts as per Seahorse analysis showed attenuation of the extracellular acidification rate (*p* < 0.05) and no significant difference in oxygen-consumption rate in SKOV3 cells. Treatment with PKM2 inhibitor suppressed ovarian cancer growth and cell migration *in vitro* and inhibited tumor growth without significant toxicity in a xenograft study. PKM2 inhibition disturbed Warburg effects and inhibited ovarian cancer cell growth. Targeting PKM2 may constitute a promising therapy for patients with ovarian cancer, and clinical trials involving shikonin are warranted.

## Introduction

Ovarian cancer is among the most common gynecologic cancers, with an estimated 21,290 cases resulting in 14,180 deaths in the United States in 2015 [1]. This is a leading cause of death from gynecologic cancers, because the symptoms are usually non-specific until the tumor has metastasized, resulting in two-thirds of cases being diagnosed at advanced stages. Ovarian cancer treatment requires intensive surgical intervention and further adjuvant chemotherapies [2]; however, recurrence and drug resistance frequently occur, especially in patients in advanced stages. Despite significant surgical advances, changes in chemotherapeutic regimens, and the development of targeted therapy, <40% of women with ovarian cancer are cured [3]. Currently, ovarian malignancy represents one of the greatest clinical challenges, and new therapeutic strategies are needed.

Dysregulated metabolism constitutes a new hallmark of cancer, and clinical evidence shows that metabolic programming associated with tumors is related to cancer outcomes. Conceptual progress resulted in the addition of an emerging field related to reprogramming energy metabolism, and focus on metabolic pathways in cancer cells has become a trend of considerable interest [4]. The Warburg effect is a metabolic characteristic associated with cancer cells, where glycolysis rather than glucose oxidation is favored to yield lactate [5, 6]. Studies showed that certain agents, such as metformin and lovastatin, can inhibit cancer cell growth by targeting and disrupting cancer cell metabolism [7–9]. Recent reports established a relationship between oncogenic pathways and tumor metabolism [10]; however, if tumor metabolism is a key to cancer progression, knowledge of the metabolic state of cancer cells is needed. Metabolic pathways associated with ovarian cancer cells remain unclear, and studies focused on ovarian cancer and its energy programming are rare. Our previous research demonstrated that niclosamide administration disrupts multiple metabolic pathways, including oxidative phosphorylation, glycolysis, and fatty acid biosynthesis, in ovarian stem cells [11]. Therefore, interfering with metabolic pathways in ovarian cancer cells may represent a novel therapeutic approach.

Aerobic glycolysis is a hallmark of the Warburg effect and is vital for cancer cell survival [12]. Pyruvate kinase M2 (PKM2) is a key enzyme regulating glycolysis and oxidative phosphorylation. PK catalyzes the last step of glycolysis, transferring the phosphate from phosphoenolpyruvate to adenosine diphosphate, thus yielding adenosine triphosphate (ATP) and pyruvate. Recently, PKM2 was reported to be a major isoform expressed in different cancer cells [13, 14]. Given that PKM2 is an important metabolic enzyme associated with cancer cells, targeting PKM2 constitutes an appealing therapeutic strategy. In this study, we investigated the clinical relevance of PKM2 in ovarian cancer and tested the therapeutic potential of PKM2 inhibitors.

## Materials and methods

### Reagent and cell lines

Shikonin powder (for follow-up experiments) was purchased from Sigma-Aldrich (St. Louis, MO, USA) and was dissolved in dimethyl sulfoxide (DMSO). IOSE, CP70, and SKOV3 cells were maintained in Roswell Park Memorial Institute (RPMI)-1640 medium (Gibco, Rockville, MD, USA). All media were supplemented with 10% fetal bovine serum (Invitrogen, Carlsbad, CA, USA) and 100 IU/mL penicillin-streptomycin at 37°C under a humidified atmosphere containing 5% CO_2_.

### Patients and clinical samples

This study was approved by the Institutional Review Board of the Tri-Service General Hospital (TSGH IRB No: 2-103-05-026). Tissue samples were collected with the informed consent of patients at the Tri-Service General Hospital, National Defense Medical Center in Taipei, Taiwan. Tumor grades were classified as well-differentiated [nuclear grade 1 (G1)], moderately differentiated [nuclear grade 2 (G2)], or poorly differentiated carcinoma [nuclear grade 3 (G3)]. The clinicopathological characteristics of patients were recorded by the data managers of the Gynecologic Oncology Center. Age, pre- and post-treatment serum CA125 concentrations, the International Federation of Gynecology and Obstetrics stage, histologic grade, recurrence, and survival status were recorded. Recurrence was defined as a measurable regrowth of the tumor (i.e., the patient had detectable disease following cytoreductive surgery and chemotherapy) or a serum CA-125 concentration more than twice the value of the upper limit of normal.

### Tissue microarray and immunohistochemistry (IHC)

Tissue microarrays comprised 88 epithelial ovarian cancer (EOC) samples, including 61 serous cystadenocarcinomas, 13 mucinous cystadenocarcinomas, seven endometrioid adenocarcinomas, seven clear-cell carcinomas, and 18 histologically benign ovarian tumors (seven serous cystadenomas and 11 mucinous cystadenomas).

Tissue-microarray sections were dewaxed in xylene, rehydrated in alcohol, and immersed in 3% hydrogen peroxide for 10 min to suppress endogenous peroxidase activity. Antigen retrieval was performed by heating each section at 100°C for 30 min in 0.01 M sodium citrate buffer (pH 6.0). After three 5-min rinses in phosphate-buffered saline (PBS), the sections were incubated for 1 h at room temperature with a rabbit polyclonal anti-human PKM2 antibody (Aviva Systems Biology, San Diego, CA, USA) diluted 1:100 in PBS, and bound antibodies were detected using a streptavidin-biotin-peroxidase system (Dako, Glostrup, Denmark) and 3,3´-diaminobenzidine substrate chromogen solution (Dako). Slides were counterstained with hematoxylin and analyzed using light microscopy. Control samples were processed similarly, with the exception of the omission of the primary antibody. For the evaluation of immunoreactivity and histological appearance, all tissue-microarray slides were examined and scored by two pathologists. Two pathologists screened the histologic sections and selected areas of representative tumor cells, with one tissue core (2 mm in diameter) taken from each of the representative tumor samples and placed in a new recipient paraffin block. The intensity of PKM2 immunostaining in individual cells was scored according to the following scale: 0 (no staining), 1 (weak intensity), 2 (moderate intensity), or 3 (strong intensity). The percentage of cells with positive PKM2 immunostaining at each intensity was estimated from zero to 100. The absolute value of the proportion of cells at each intensity level was multiplied by the corresponding intensity value, and these products were added to obtain an immunostaining score ranging from zero to 300.

### Cell viability assay

Cells were seeded onto a 96-well plate (1000 cells per well) for 24 h, followed by treatment with chemotherapeutic drugs for 72 h. We subsequently evaluated cell viability using the CellTiter-Glo luminescent cell viability assay (ATP assay; Promega, Annandale, NSW, Australia). Briefly, the ATP reagent (0.01 μM) was added to 100 μL of medium containing cells in each well of a 96-well plate, and the intensity of luminescence was measured 10 min after addition of the reagent.

### Scratch-wound-healing assay

For the scratch-wound-healing assay, CP70 and SKOV3 cells were grown in complete growth medium to 90% confluency. A 3-mm wound was introduced across the diameter of each plate, and cell migration was observed by microscopy 16 h later and analyzed objectively to observe the healing condition of the cell scratches.

### Colonogenic assay

Cells were trypsinized and resuspended in 1.5 mL of 0.35% agarose; the suspension was then poured onto a layer of 1.5 mL of 0.5% agarose in 35-mm tissue culture dishes. After 3–4 weeks, the cells were stained with a solution containing 0.005% crystal violet, 1.9% formaldehyde, and 0.15 M NaCl for 30 min. After washing and drying, colonies > 1 mm were counted.

### Measurement of the metabolic rates of glucose and lactate

Cells were cultured in 24-well plates at 3 × 10^5^ cells/well. After attachment, the culture medium was replaced, and cells were treated with shikonin or vehicle (0.1% DMSO) for 5 h and 7 h. The culture medium was collected, and the concentrations of glucose (hexokinase) and lactate (oxidation of lactate) in the medium were measured using a Union Clinical Laboratory kit with Dimension RXL (Siemens, Munich, Germany). Glucose consumption and lactate production were calculated from the different time points between the concentrations in the medium at the beginning and after an appropriate culture time.

### Seahorse XF24 analysis of the oxygen-consumption rate (OCR) and glycolysis

Baseline measurements of the OCR were measured using the oxygen concentration change and extracellular acidification rate (ECAR) according to changes in pH before the wells were injected with shikonin (final concentration: 3 μM). CP70 and SKOV3 cells were cultured on Seahorse XF24 plates to a density of 2 × 10^3^ cells/well, and each well was filled with unbuffered RPMI medium (pH 7.4) and incubated at 37°C before the experiment. The OCR reading represented oxygen consumption, whereas ECAR represented lactate production and the glycolytic index.

### Xenograft models

NOD/SCID mice were purchased from National Taiwan University, and all procedures were approved by the Laboratory Animal Care and Use Committee of the National Defense Medical Center. For studies of tumor xenografts, SKOV3 cells were suspended in 100 μL of Matrigel and injected subcutaneously into NOD/SCID mice. To assess the effects of treatment with the compounds identified, female NOD/SCID mice (6-weeks old) were housed under pathogen-free conditions at the Animal Center of the National Defense Medical Center. Treatment with compounds was initiated 24 h after tumor injection, and animals were administered vehicle (PBS) or shikonin (10 mg/kg) intraperitoneally 5 days per week for 3 weeks. Animals were monitored daily and clinical examination was undertaken at least twice daily. At the experimental endpoints of shikonin and vehicle treatments, mice that developed respiratory distress, signs of suffering or pain, abdominal swelling due to ascites or solitary tumor masses greater than 10% of body weight were humanely euthanized using CO_2_ and cervical dislocation according to the University of South Florida IACUC guidelines. Groups of mice were killed after 4 weeks, and the fat pads were analyzed for the presence of tumor outgrowth.

### Micro-positron emission tomography (PET) imaging and analysis

Both groups of NOD/SCID mice were injected with 0.15 mCi to 0.2 mCi (5.55–7.4 MBq) of [^18^F]-2-deoxy-2-fluoro-D-glucose (^18^F-FDG) via the tail vein. Following ^18^F-FDG injection (30 min), the mice were anesthetized, and PET-statistic scanning with BIOPET105 (Bioscan, Inc., Washington, DC, USA) was performed. PET images were acquired for 30 min, and the energy window was set at between 250 keV and 700 keV. Three-dimensional ordered subset expectation maximization was employed to reconstruct the images. All of the imaging procedures were performed at baseline in the National Defense Medical Center Laboratory Animal Center qualified by the Association for Assessment and Accreditation of Laboratory Animal Care International (AAALAC 2007). Imaging data were analyzed using Amide software (Loening and Gambhir, 2003). Tumor volume was quantified by calculating the specific uptake value, which represents the ratio of the ^18^F-FDG levels in a volume of interest (typically of a focal tumor) to the average ^18^F-FDG levels in the entire body.

### Statistical analysis

The mean and the standard error of the mean are reported. Data were compared using two-tailed and Student’s *t* tests. Differences were considered significant at *p* < 0.05.

## Results

### Clinicopathological correlations of PKM2

**Fig 1:**
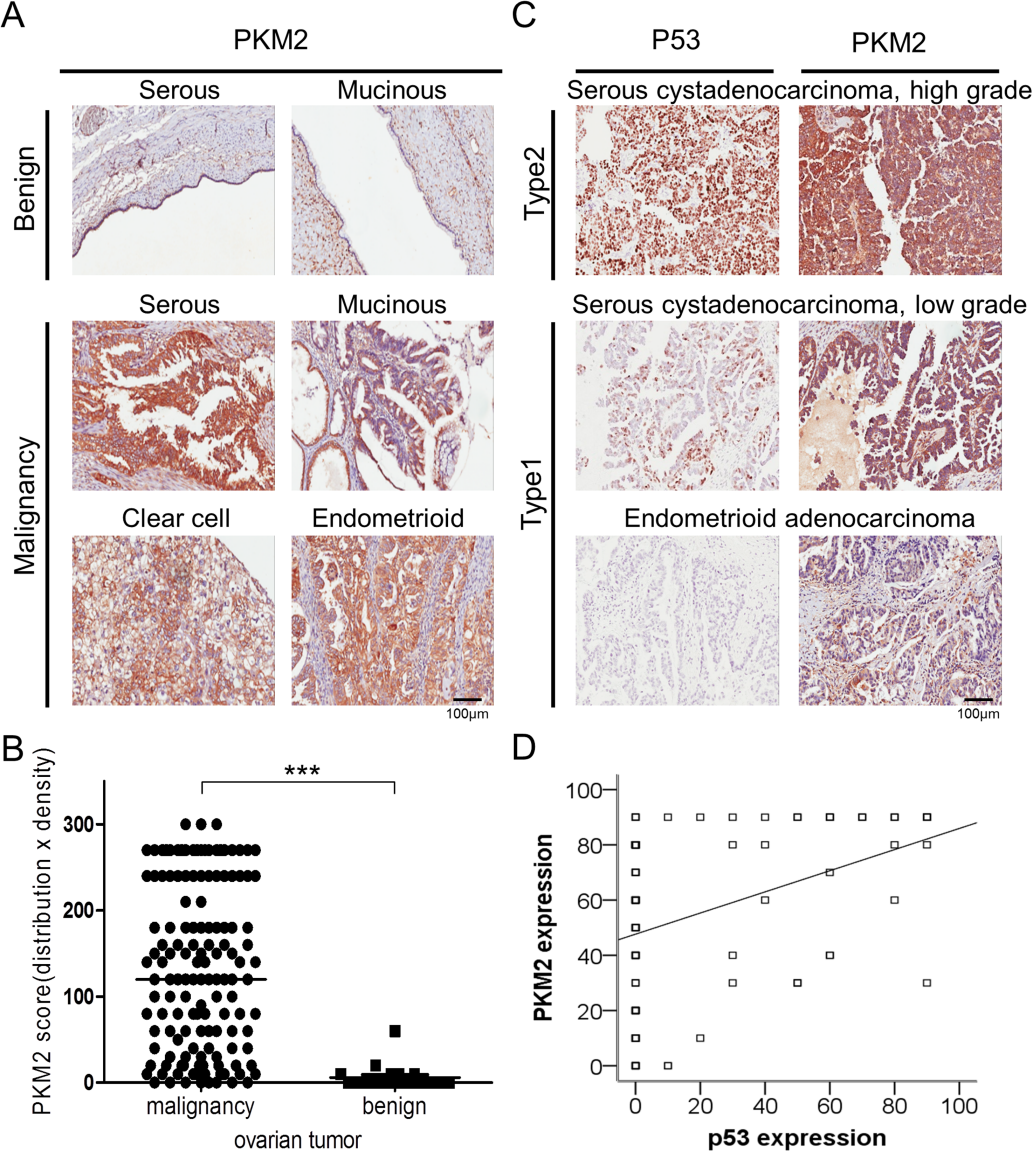
Expression and distribution of PKM2 in different histological types of epithelial ovarian tumor. (A) Proportion of PKM2 expression in cells of different histologic types of benign and malignant ovarian tumor. (B) Representative differential PKM2 expression in benign and malignant groups of epithelial ovarian tumor. *p < 0.001. (C) Proportion of p53 and PKM2 expression in cells of different histologic models of ovarian cancer. (D) Spearman's rho showed moderate correlations between PKM2 and p53 expression. PKM2, Pyruvate kinase M2.

To determine links between PKM2 expression and clinical characteristics of ovarian cancer, we performed IHC analysis using ovarian cancer tissue microarrays (Fig 1A). Patients were divided into two groups: PKM2 high (score ≥ 50) and PKM2 low (score < 50; Table 1). PKM2 expression was significantly associated with the malignancy group (Fig 1B; *p <* 0.001), histologic type (*p =* 0.008), and pathogenesis model (Table 1; *p <* 0.001). To further evaluate the relationship between p53 and PKM2 expression, we determined the association of PKM2 and p53 using immunostaining (Fig 1C), finding moderate correlation between p53 and PKM2 expression (Spearman's rho = 0.42; *p* < 0.001; Fig 1D).

**Table 1:** Clinicopathological features.

	PKM2 expression				
	<50		≥50		*P* value
Patients, n	37		51		
Age (years)					0.36
Range	16–79		24–81		
Mean ± SEM	51.7 ± 2.9		54.5 ± 1.7		
Stage, n (%)					0.05
I, II	17	(56.7)	11	(43.3)	
III, IV	20	(34.5)	35	(65.5)	
Grade, n (%)					0.66
Low (G1, G2)	17	(44.7)	21	(55.3)	
High (G3)	20	(40.0)	30	(60.0)	
Histologic type, n (%)					**0.008**
Serous type	20	(32.8)	41	(67.2)	
Other types	17	(63.0)	10	(37.0)	
Pathogenesis model, n (%)					**<0.001**
Type I	20	(69.0)	9	(31.0)	
Type II	17	(28.8)	42	(71.2)	
Significance in bold (P < 0.05)					
Histologic type, n (%)					0.01 ^a^
SC	20	(32.8)	41	(67.2)	
MC	13	(72.2)	5	(27.8)	
EC	2	(66.7)	1	(33.3)	
CCC	2	(33.3)	4	(66.7)	

^a^ Fisher’s exact test

**Fig 2:**
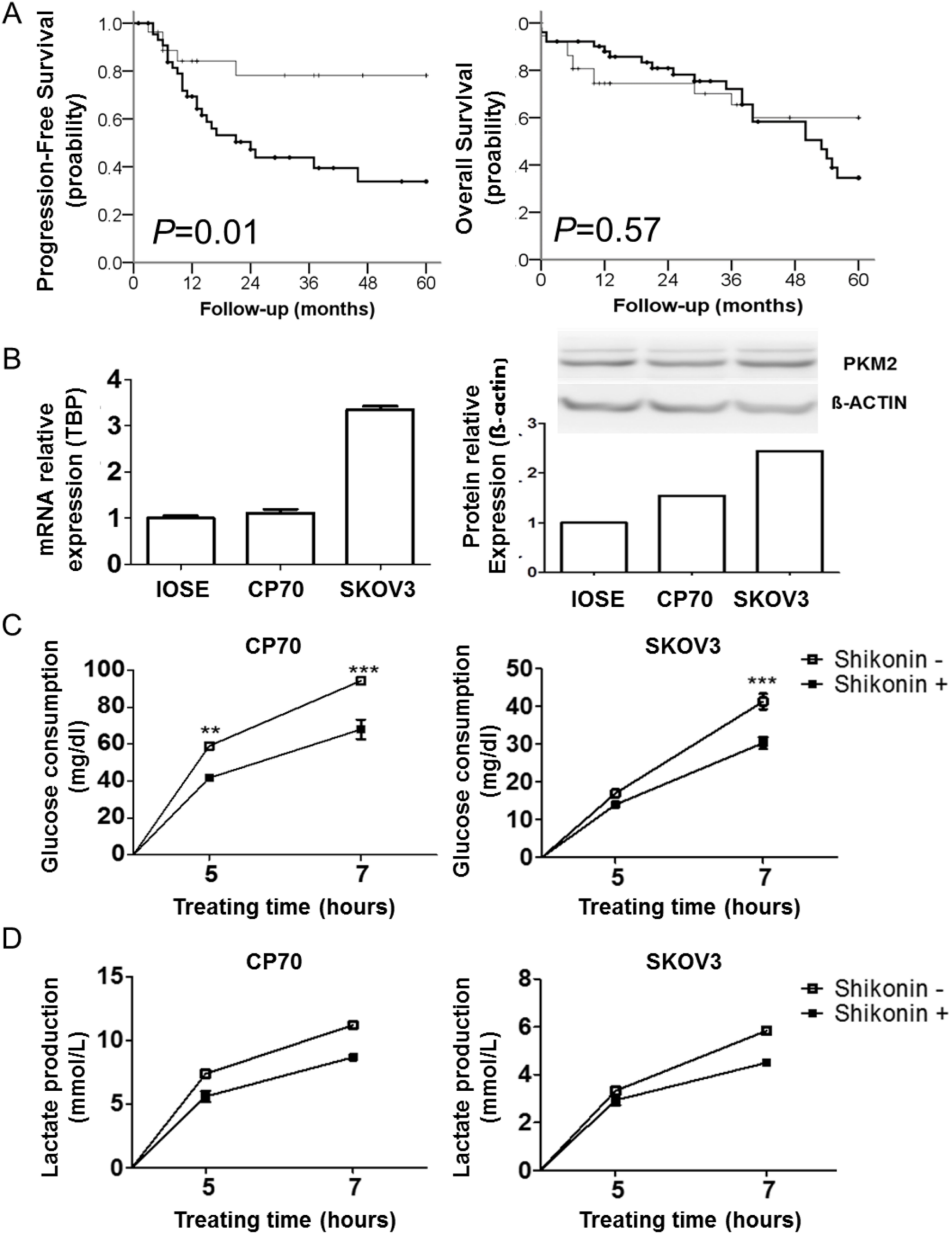
PKM2 expression decreases the survival of patients and PKM2 inhibition disrupts the glycolytic pathway in ovarian cancer cells. (A) Kaplan–Meier analysis of the probability of progression-free survival (*p* = 0.01) (left) and overall survival (*p* = 0.057) (right) in patients with ovarian cancer, stratified according to PKM2 expression (*n* = 88). (B) Expression of PKM2 in different ovarian cancer cell lines. (C and D) Time-dependent inhibition of glucose consumption (**p<0.01,***p<0.001) and lactate production in CP70 and SKOV3 cells following shikonin treatment. PKM2, Pyruvate kinase M2.

There were no significant associations between PKM2 expression and age, stage, or nuclear grade (Table 1). Patients with higher PKM2 expression exhibited poor progression-free survival rates (Fig 2A; *p =* 0.01), but PKM2 expression was not associated with overall survival rates (Fig 2A; *p =* 0.57). Multivariate analysis revealed that higher PKM2 levels conferred a hazard ratio of progression-free survival of 2.02 (95% confidence interval: 0.70–5.85; Table 2). These data suggested that PKM2 expression was important to ovarian cancer cell survival and progression.

**Table 2:** Univariate and multivariate Cox regression analysis for progression-free and overall survival in patients with ovarian cancer.

	Progression-free Survival		Overall Survival	
Variable	Univariate analysis crude HR (95% CI)	Multivariate adjusted HR (95% CI) ^a^	Univariate analysis crude HR (95% CI)	Multivariate adjusted HR (95% CI) ^d^
Age (years)	1.03 (0.99–1.06)	-	1.05 (1.02–1.08) ^c^	1.03 (1.00–1.06) ^c^
PKM2 expression ^b^				
Low	1.00 (reference)	1.00 (reference)	1.00 (reference)	1.00 (reference)
High	3.22 (1.22–8.48) ^c^	2.02 (0.70–5.85)	1.19 (0.59–2.41)	0.68 (0.33–1.40)
Stage				
I, II	1.00 (reference)	1.00 (reference)	1.00 (reference)	1.00 (reference)
III, IV	16.07 (3.77–68.55) ^c^	21.71 (3.36–140.33) ^c^	26.25 (3.58–192.48) ^c^	13.23 (1.73–100.86) ^c^
Grade				
Low	1.00 (reference)	1.00 (reference)	1.00 (reference)	1.00 (reference)
High	4.16 (1.77–9.78) ^c^	1.90 (0.77–4.68)	13.80 (4.15–45.87) ^c^	5.31 (1.56–18.07) ^c^
Histologic type				
Serous type	3.54 (1.07–11.74) ^c^	0.28 (0.05–1.46)	1.67 (0.69–4.04)	-
Other types	1.00 (reference)	1.00 (reference)	1.00 (reference)	-

Abbreviations: HR, hazard ratio; CI, confidence interval

^a^ The analysis adjusted for PKM2 expression, stage, grade, and histologic type

^b^ The low expression of PKM2 regarding survival is represented as <50, and the high expression of PKM2 regarding survival is represented as > = 50.

^c^ Significantly correlated with outcome, *p* < 0.05

^d^ The analysis adjusted for age, PKM2 expression, stage, and grade

### PKM2 inhibition disrupts metabolism and attenuates malignant phenotypes in ovarian cancer cells

We next investigated the functional effect of PKM2 in ovarian cancer cell lines. By using RT-PCR and western blot, we verified that PKM2 was overexpressed in all cell lines tested, especially SKOV3 cells (Fig 2B); therefore, CP70 and SKOV3 cells were chosen for further studies.

**Fig 3:**
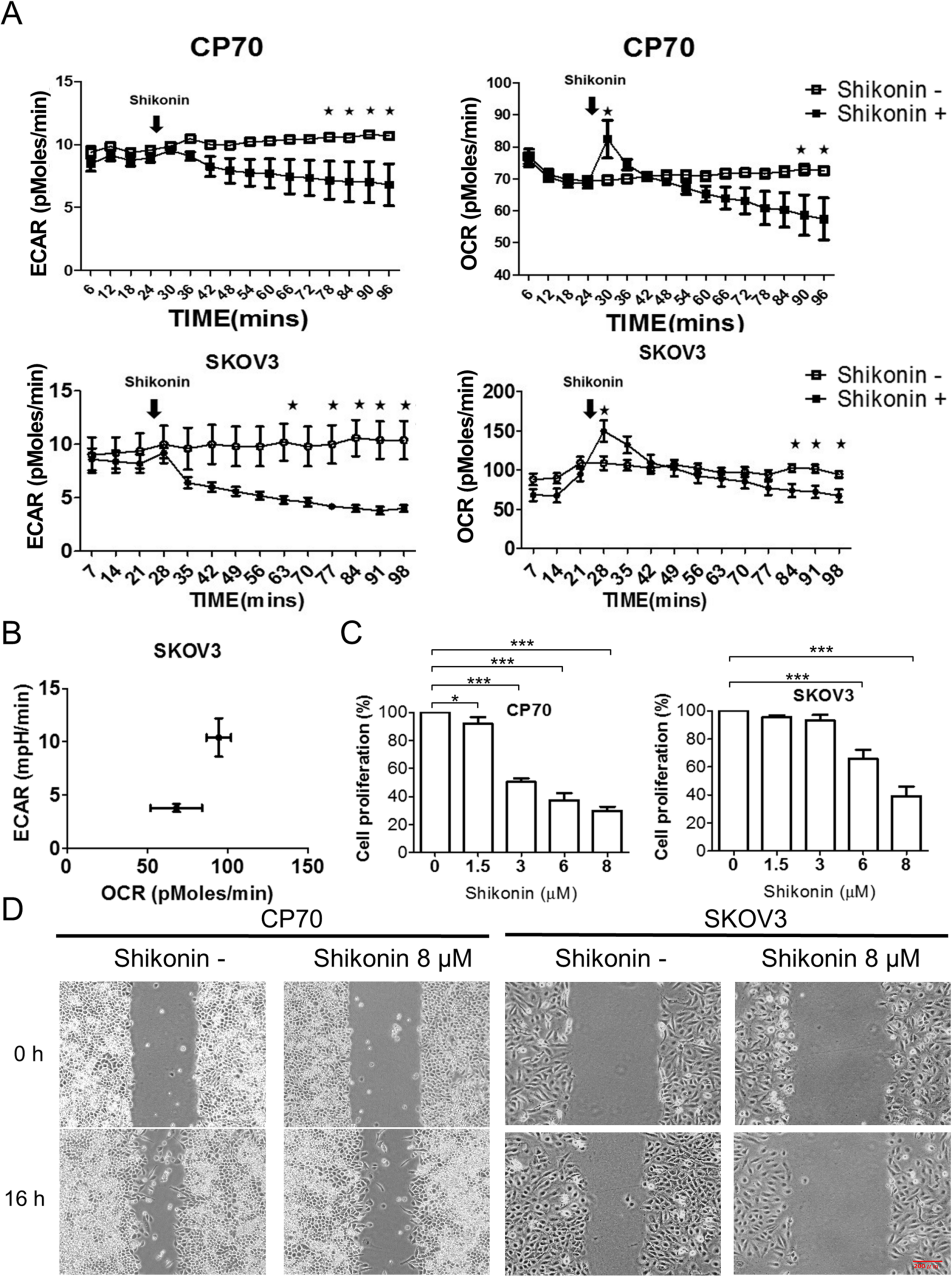
PKM2 inhibition disrupts the energy shift in ovarian cancer cells and suppresses ovarian cancer cell growth. (A) The ECAR according to Seahorse analysis decreased following shikonin treatment in SKOV3 and CP70 cells (*p<0.05). (B) PKM2 inhibition induced a shift in OCR/ECAR in SKOV3 cells. (C) Dose-dependent inhibition of cancer cell growth in CP70 and SKOV3 cells following shikonin treatment. (D) Migration assay involving CP70 and SKOV3 cells following shikonin treatment. ECAR, extracellular acidification rate; OCR, oxygen-consumption rate; PKM2, Pyruvate kinase M2.

To determine the metabolic effect of PKM2 interference on ovarian cancer cells, we measured glycolysis, glucose consumption, and lactate production following treatment with the PKM2 inhibitor shikonin [15]. Glucose consumption was significantly decreased (*p* < 0.001) and lactate production also decreased following shikonin treatment as compared with levels observed in control groups (Fig 2C and 2D). To determine the effect of PKM2 inhibition on ovarian cancer energy shift, we used the Seahorse XF24 assay to analyze ECAR and OCR. Ovarian cancer cells showed a significant decrease in ECAR (*p* < 0.05) and a temporary compensatory increase, but gradual decrease in OCR following treatment with PKM2 inhibitor in CP70 and SKOV3 cells (Fig 3A). The energy shift revealed blockage of ECAR and no significant difference between the OCR in SKOV3 (Fig 3B) and CP70 cells (S1 Fig). Additionally, shikonin administration also inhibited ovarian cancer cell proliferation (Fig 3C), migration (Fig 3D and S2 Fig), and colony formation (S3 Fig). These results confirmed that PKM2 inhibition caused a metabolic disturbance and inhibited malignant phenotypes in ovarian cancer cells.

### PKM2 inhibition reduces xenograft tumor growth

**Fig 4:**
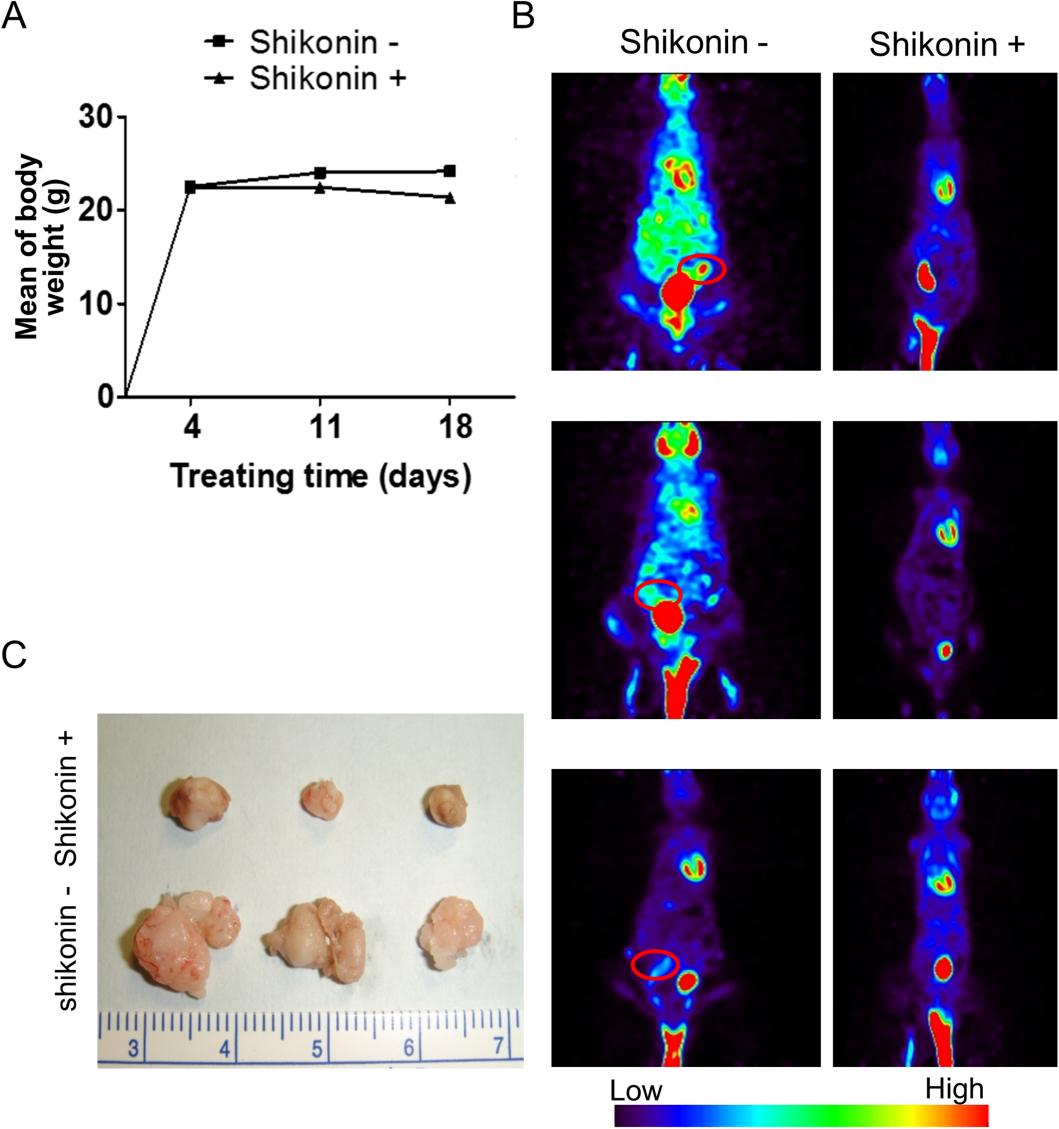
PKM2 inhibition attenuates tumor growth in the tumor xenografts. (A) Treatment with shikonin did not cause differences in body weight loss between the treatment and control groups. (B) Representative micro-PET images of xenograft mice in the shikonin-treated group as compared with the control group. (C) Shikonin treatment inhibited tumor formation *in vivo* following injection of SKOV3 ovarian cancer cells. PET, positron emission tomography; PKM2, Pyruvate kinase M2.

**Fig 5:**
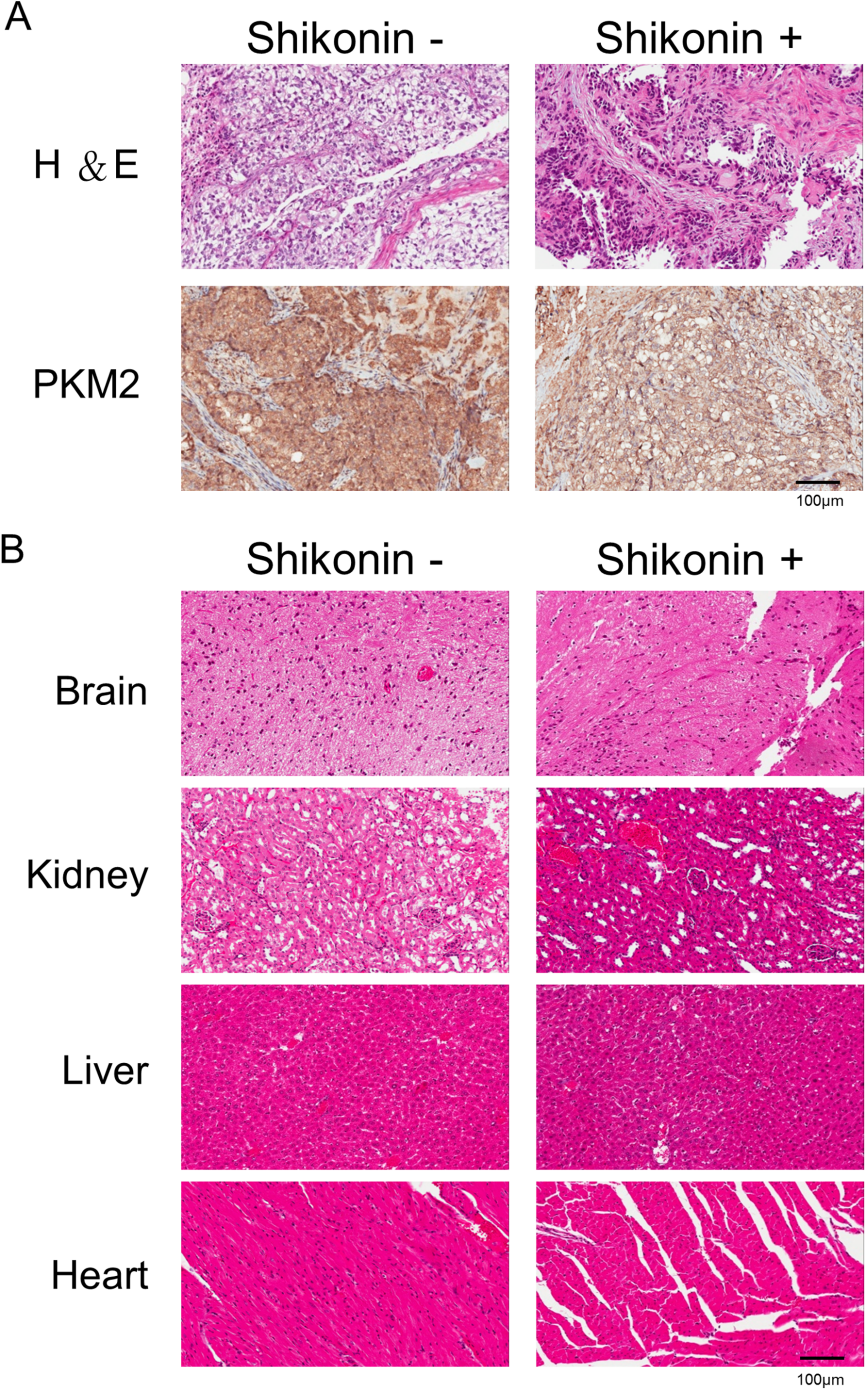
Evaluation of the cytotoxicity of PKM2-inhibitor administration in mice. (A) H&E and IHC stains of mouse tumors following shikonin treatment. (B) Shikonin treatment did not cause apparent pathologic abnormalities in the brain, kidney, liver, or heart according to H&E staining. H&E, hematoxylin and eosin; IHC, immunohistochemistry; PKM2, Pyruvate kinase M2.

According to *in vitro* results, we assessed the therapeutic effects of shikonin *in vivo*. SKOV3 cells were injected in mice intraperitoneally, and shikonin was administrated intraperitoneally for 5 days over the course of 1 week. We subsequently treated mice with shikonin (10 mg/kg) intraperitoneally 5 days per week for 3 weeks. In the vesicle control and shikonin-treated group, one mouse from each group died prior to being euthanized due to signs of suffering or pain. The remaining mice tolerated the therapy well and did not exhibit apparent body weight loss at the shikonin testing doses used (Fig 4A). Micro-PET scans of tumor growth following shikonin treatment for 3 weeks showed decreases in tumor growth in the shikonin-treated group (Fig 4B, red circle). At 4 weeks, the mice were sacrificed (n = 6). The sizes of tumors in the presence (n = 3) or absence of treatment (n = 3) with shikonin are shown in Fig 4C. Tumor size measurements of the treated group were reduced as compared with those of the control group (Fig 4C). In the vesicle control group, the maximal tumor measured 1.2 cm in diameter. All shikonin-treated mice (n = 3) showed a decrease in tumor burden. This confirmed that shikonin inhibits tumor growth and reduces tumor weight. Additionally, PKM2 expression according to IHC staining also showed a significant reduction following shikonin treatment (Fig 5A). To further evaluate the toxicity associated with administration of the PKM2 inhibitor *in vivo*, we performed pathological analyses. As shown in Fig 5B, we found no pathologic changes in different organs, including the brain, kidney, liver, and heart, based on microscopic examination. These results supported the hypothesis that PKM2 inhibition can reduce ovarian cancer cell growth without significant cytotoxicity.

## Discussion

Cancer metabolism is a focus of cancer therapy. Conceptual progress resulted in the addition of an emerging field related to reprogramming energy metabolism, and focus on metabolic pathways in cancer cells has become a trend of considerable interest [4]. Unlike normal differentiated cells, proliferating cancer cells exhibit different metabolic requirements. The Warburg effect indicates that many cancer cells produce energy by glycolysis, followed by lactic acid fermentation, with shifts from oxidative phosphorylation to aerobic glycolysis constituting the best characterized metabolic phenotypes associated with cancer [16]. Carbon is diverted into biosynthetic pathways required for high rates of cell proliferation, and a recent study demonstrated that cancer cells require specific metabolic programming for proliferation and survival [17]. This study revealed altered metabolism in ovarian cancer cells following the inhibition of PKM2 expression, indicating that dysregulated PKM2 may be a potential therapeutic target in ovarian cancer.

Targeting cancer metabolism is an emerging field in drug discovery. However, studies related to energy production associated with ovarian cancer are limited. A recent study demonstrated that omental adipocytes supply fatty acid as an energy source and promote ovarian cancer progression, suggesting unique pathways involving lipid metabolism in ovarian cancer [18]. Our previous showed that the anthelmintic drug niclosamide targets cancer stem cells and disrupts multiple metabolic pathways, including oxidative phosphorylation, glycolysis, and fatty acid biosynthesis, in ovarian and breast tumor-initiating cells [11, 19]. Since the recent paradigm shift from a stochastic model to a cancer stem cell model of tumorigenesis [20]. The discussion of cancer stem cells and their metabolic state is rare. A recent study also indicated that PKM2 promotes the stemness of breast cancer cells [21]. In the current study, we demonstrated the effects of dysregulation of PKM2, an important regulator of glycolysis and oxidative phosphorylation in ovarian cancer cells. Cancer cells exhibit metabolic changes depending on genetic alterations, and subsequently modify metabolic pathways to increase nutrition and cancer growth. Interference of this metabolic program is a potential strategy for cancer therapy [22, 23]. In mammalian cells, there are two PK genes: one encodes PKM1 and PKM2, and the other encodes liver-type PK (PKL) and PKR [24]. Different levels of PKM activities lead to different outcomes, with low PKM2 activity promoting conversion of pyruvate to lactate and high PKM1 or PKM2 activity promoting pyruvate conversion to acetyl-CoA [6, 14, 25]. Recently, studies demonstrated that PKM2 promotes tumorigenesis and aerobic glycolysis in many cancers [11, 13, 26], and several other studies revealed PKM2 as a key enzyme that regulates the Warburg effect and necessary for energy production to support tumor growth [13]. Because PKM2 contributes to the malignant phenotype of many cancers, focusing on PKM2 function as a regulator of tumor metabolism might be a potential strategy for targeting ovarian cancer’s Achilles heel.

Here, we chose shikonin as a PKM2 inhibitor. Shikonin is a novel compound isolated form Chinese herbal agents (Zicao) and has been investigated as a potential anticancer drug for use in various aspects of cancer treatment [22, 23, 27]. The mechanism associated with shikonin-related anticancer activity is poorly understood. A recent study demonstrated that shikonin inhibits the growth of SKOV3 cells, induces their apoptosis, and reduces their migration through inhibiting the phosphorylation of Src and FAK; these results are consistent with those reported in our study [28]. Another study also demonstrated the application of shikonin-loaded antibody-armed nanoparticles for targeted therapy in ovarian cancer[29]. Based on these findings, we propose that shikonin may be a novel small molecule that could be used in targeted therapy for ovarian cancer. Furthermore, other studies reported that its administration inhibited ovarian cancer glycolytic metabolism, and our results indicated that targeting ovarian cancer glycolytic metabolism could be an effective strategy for cancer therapy [12,23]. Recently, shikonin was reported to inhibit PKM2, leading to suppression of cancer cell proliferation and survival [15]. Another study reported that PKM2, but not PKM1 or PKL, was bound by shikonin, resulting in inhibition of PKM2 expression and suppression of mitochondrial function in tumor cells; shikonin showed a promising selectivity toward PKM2; shikonin at concentrations that resulted in over 50% inhibition of PKM2 activity did not inhibit PKM1 and PKL[15, 30]. Other studies also reported anti-inflammatory and anti-tumor activities related to shikonin treatment, including the inhibition of tumor growth by induction of apoptosis, inhibition of DNA topoisomerase, and inhibition of angiogenesis [26, 31, 32]. Although previous studies also reported shikonin in ovarian cancer, none reported mechanistic insights. In this study, we used the Seahorse analyzer to directly analyze the effects of shikonin on oxidative phosphorylation and glycolysis in ovarian cancer metabolism. However, the limitations associated with this drug include its lack of specificity for PKM2; therefore, discovery of a more specific PKM2 inhibitor is needed for the development of more effective drugs. Here, we demonstrated that shikonin is a potent glycolysis inhibitor in ovarian cancer cells; however, further research regarding use of this inhibitor is necessary.

Our findings demonstrated that dysregulated PKM2 in ovarian cancer cells constituted a potential therapeutic target for the treatment of ovarian cancer. We feel that our results support clinical trials focused on repurposing shikonin for ovarian cancer treatment.

## Supporting information

S1 Fig PKM2 inhibition induces a shift in OCR/ECAR in CP70 cells. ECAR, extracellular aci dification rate; OCR, oxygen-consumption rate; PKM2, Pyruvate kinase M2.
(TIF)

S2 Fig PKM2 significantly inhibits the migration ability in CP70 and SKOV3 cells. PKM2, Pyruvate kinase M2. (*p<0.05,**p<0.01).
(TIF)

S3 Fig Colony formation assay for the assessment of anchorage-independent growth using CP70 and SKOV3 cells following shikonin treatment. PKM2 significantly inhibited colony formation in CP70 and SKOV3 cells.
PKM2, Pyruvate kinase M2.
(TIF)
