# Supplementary figures and images for: Pyruvate kinase M2 is a poor prognostic marker of and a therapeutic target in ovarian cancer
Source: PLoS One. 2017 Jul 28;12(7):e0182166. doi: 10.1371/journal.pone.0182166 (PMC5533430; doi:10.1371/journal.pone.0182166)

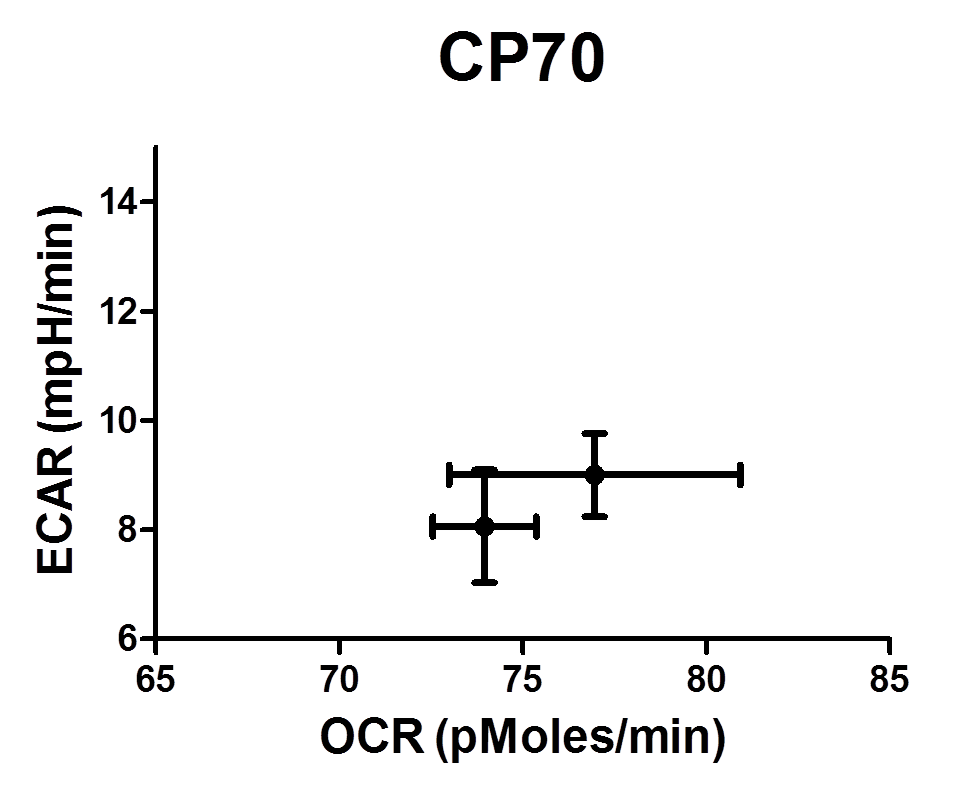

Supplement: S1 Fig — ECAR, extracellular aci dification rate; OCR, oxygen-consumption rate; PKM2, Pyruvate kinase M2. (TIF) [file pone.0182166.s001.tif]

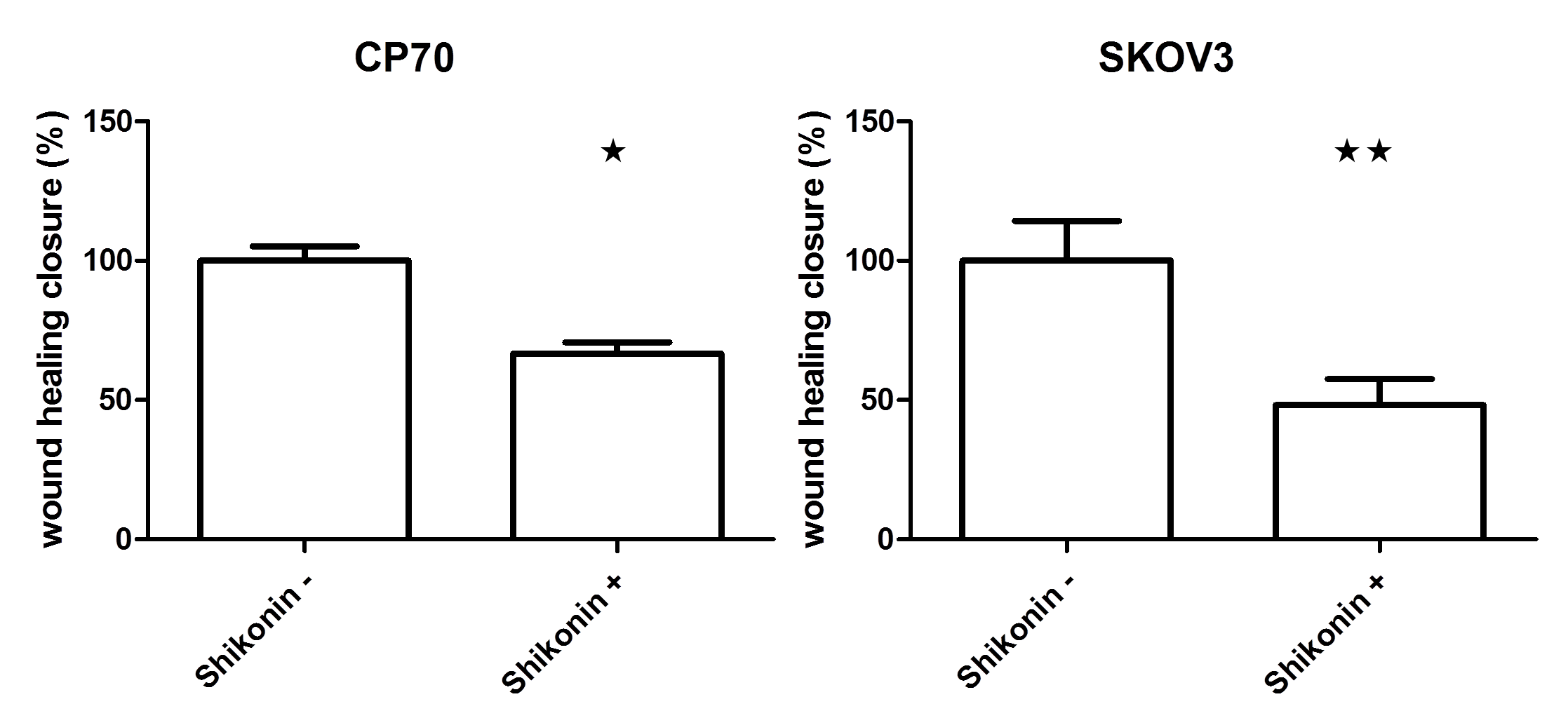

Supplement: S2 Fig — PKM2, Pyruvate kinase M2. (*p<0.05,**p<0.01). (TIF) [file pone.0182166.s002.tif]

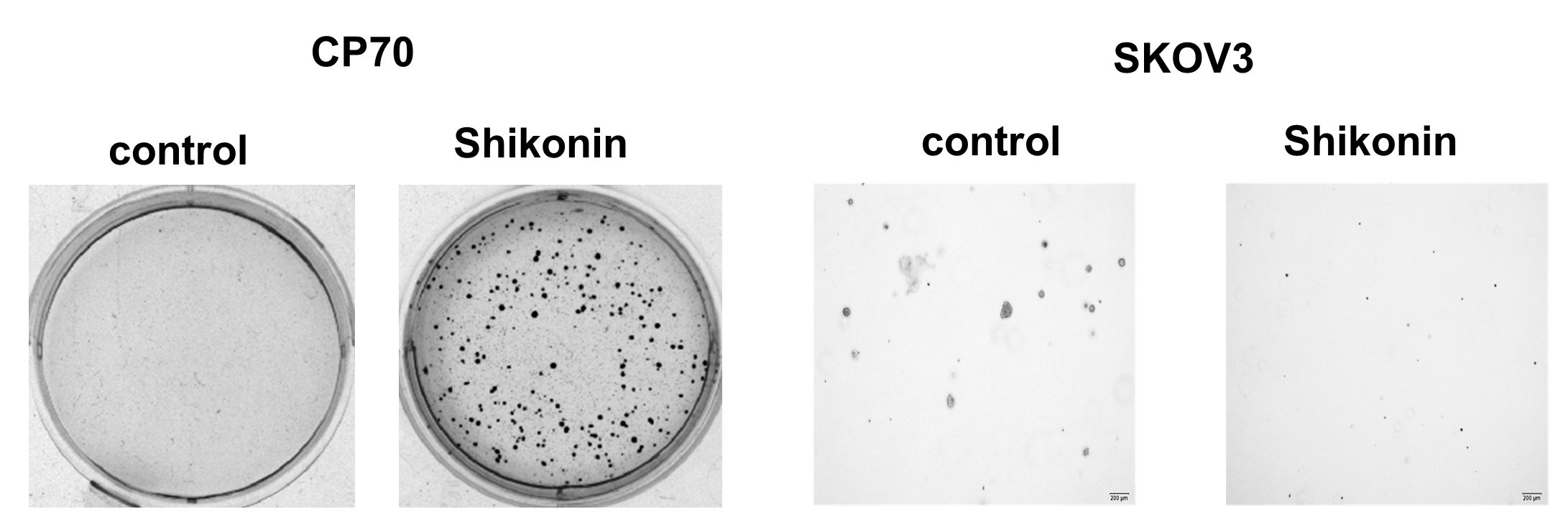

Supplement: S3 Fig — PKM2 significantly inhibited colony formation in CP70 and SKOV3 cells. PKM2, Pyruvate kinase M2. (TIF) [file pone.0182166.s003.tif]
